# Supplementary material for: The sexual behaviours of adolescents aged between 14 and 17 years involved with the juvenile justice system in Australia: A community-based survey
Source: PLoS One. 2020 Dec 28;15(12):e0243633. doi: 10.1371/journal.pone.0243633 (PMC7769256; doi:10.1371/journal.pone.0243633)
Supplement: S1 File — (DOCX) [file pone.0243633.s001.docx]

**Supplementary Figure 1a. Young people aged 14-15 years old under supervision on an average day by state and MeH-JOSH**

**
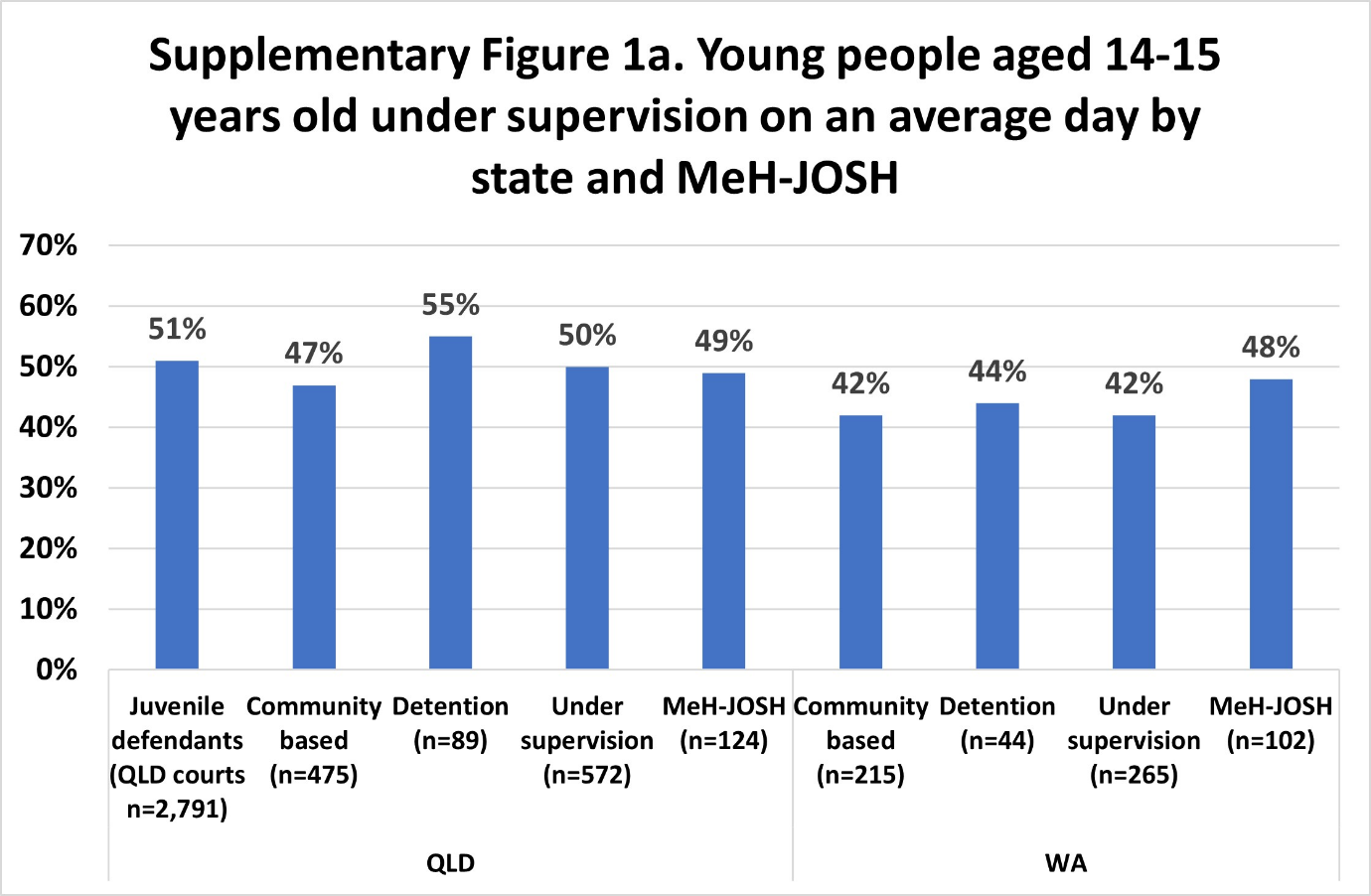
**

**Supplementary Figure 1b. Young people aged 16-17 years old supervision on an average day by state and MeH-JOSH**

**
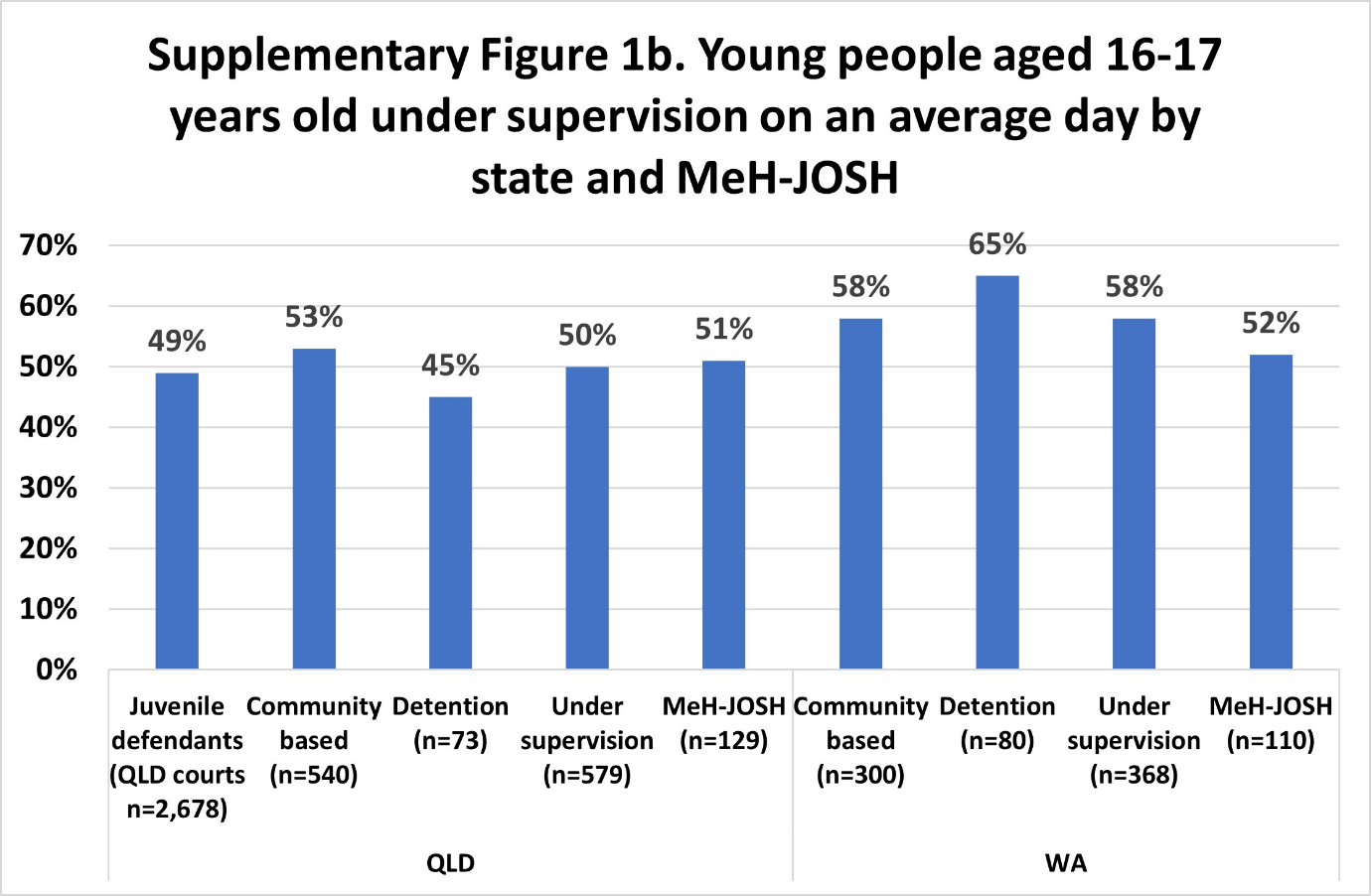
**

*Source:*

*AIHW Youth Justice Qld S132a-S132c and WA S134a-S134c. Available at:* [*https://www.aihw.gov.au/getmedia/38637613-61ed-4709-a42a-1c1b77d8138d/aihw-juv-116-state-and-territory-tables-s128-to-s143-2016-17-data-table.xlsx.aspx*](https://www.aihw.gov.au/getmedia/38637613-61ed-4709-a42a-1c1b77d8138d/aihw-juv-116-state-and-territory-tables-s128-to-s143-2016-17-data-table.xlsx.aspx)

*Children’s Court of Qld Annual Report 2016-2017*

In general, the study had an over representation of Aboriginal (Indigenous) young people when we compared it to the general Aboriginal populations in Australia and reflects the greater involvement of this population in the justice system (Supplementary Figure 2).

**Supplementary Figure 2. Aboriginal populations in Australia and MeH-JOSH**


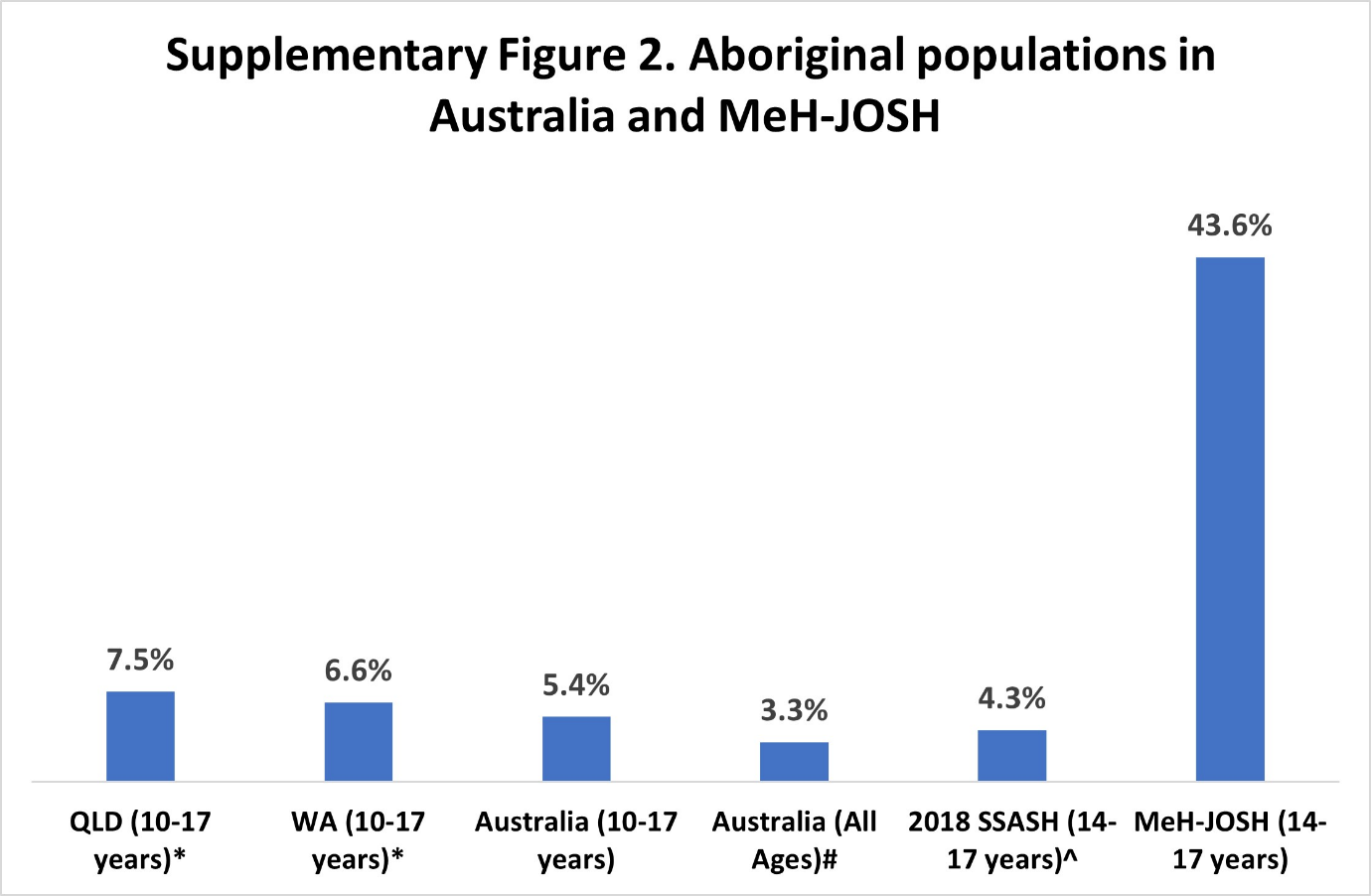


*Source:*

**AIHW Youth Justice. Table S31: Australian population aged 10–17 by Indigenous status, states and territories, December 2013 to December 2017. Available at:* [*https://www.aihw.gov.au/getmedia/993d8e77-b18e-4482-b9b9-12fe838db158/aihw-juv-128-youth-detention-population-in-Australia-2018-data-tables.xlsx.aspx*](https://www.aihw.gov.au/getmedia/993d8e77-b18e-4482-b9b9-12fe838db158/aihw-juv-128-youth-detention-population-in-Australia-2018-data-tables.xlsx.aspx)

*# Australian Bureau of Statistics (2016). 3238.0.55.001 – Estimates of Aboriginal and Torres Strait Islander Australians, June 2016. Available at: http://www.abs.gov.au/ausstats/abs@.nsf/Lookup/3238.0.55.001main+features1June%202016*

*^2018 Secondary School Student Sexual Health Survey, personal communication, C Fisher.*
