# Supplementary material for: The sexual behaviours of adolescents aged between 14 and 17 years involved with the juvenile justice system in Australia: A community-based survey
Source: PLoS One. 2020 Dec 28;15(12):e0243633. doi: 10.1371/journal.pone.0243633 (PMC7769256; doi:10.1371/journal.pone.0243633)
Supplement: Attachment — Submitted filename: Response to PLoSOne.docx [file pone.0243633.s002.docx]

**Journal requirements:**

**When submitting your revision, we need you to address these additional requirements.**

**1. Please ensure that your manuscript meets PLOS ONE's style requirements, including those for file naming. The PLOS ONE style templates can be found at**
[**http://www.journals.plos.org/plosone/s/file?id=wjVg/PLOSOne_formatting_sample_main_body.pdf**](http://www.journals.plos.org/plosone/s/file?id=wjVg/PLOSOne_formatting_sample_main_body.pdf) **and** [**http://www.journals.plos.org/plosone/s/file?id=ba62/PLOSOne_formatting_sample_title_authors_affiliations.pdf**](http://www.journals.plos.org/plosone/s/file?id=ba62/PLOSOne_formatting_sample_title_authors_affiliations.pdf)

**2. You indicated that you had ethical approval for your study. In your Methods section, please ensure you have also stated whether you obtained consent from parents or guardians of the minors included in the study or whether the research ethics committee or IRB specifically waived the need for their consent. In this manuscript parental consent is only referred to for those minors recruited from the Children's Court. Additionally, minors were recruited from "youth mental health service centres". Please confirm that this vulnerable population were determined to be capable of providing consent.**

We have added to the manuscript:

“Due to the nature of this population and from our experiences of conducting surveys among young offenders in Australia[3], we expected that a high proportion of young people would not have a good relationship with their parent(s) or with the adults responsible for them. Thus human research ethics approval was sought and given for young people recruited in the survey to be treated as mature minors.[14] Nevertheless, all young people approached were asked by recruiters if we could contact their parents or guardians for permission to allow them to participate in the survey. Almost all respondents refused us contact or to give any contact details of their parents or guardians preferring to give consent themselves. Recruiters were required to administer a Gillick Competency checklist to ensure that all respondents met the criteria of a mature minor.”

**Furthermore, the site of recruitment was not included as a potentially confounding factor in the statistical analyses. Please justify why this was not performed.**

Site of recruitment was not a confounding factor for age and gender although it was significant for Aboriginality (p<0.05).

**3. Please include additional information regarding the survey or questionnaire used in the study and ensure that you have provided sufficient details that others could replicate the analyses. For instance, if you developed a questionnaire as part of this study and it is not under a copyright more restrictive than CC-BY, please include a copy, in both the original language and English, as Supporting Information. Additionally, please refer to any post-hoc corrections made during your statistical analysis for multiple comparisons. Please justify the reasons if these were not performed.**

Parts of the questionnaire (MINI KID 6.0) are under copyright restrictions (D. Sheehan).

This paper focuses on the descriptive analysis of the MeH-JOSH and data obtained from other studies. Multivariate analyses were not performed for the main tables in this paper.

Additional Editor Comments (if provided):

The reviewers identified several ways to make the paper clearer to the reader. I am in agreement with both reviewers that this is a useful study that will likely ultimately merit publication, but the authors will need to accomplish all of the revisions identified below in order to meet the publication criteria.


[Note: HTML markup is below. Please do not edit.]

Reviewers' comments:

Reviewer's Responses to Questions

**Comments to the Author**

**1. Is the manuscript technically sound, and do the data support the conclusions?

The manuscript must describe a technically sound piece of scientific research with data that supports the conclusions. Experiments must have been conducted rigorously, with appropriate controls, replication, and sample sizes. The conclusions must be drawn appropriately based on the data presented.

Reviewer #1: Yes

Reviewer #2: No**

**2. Has the statistical analysis been performed appropriately and rigorously?

Reviewer #1: Yes

Reviewer #2: No**

**3. Have the authors made all data underlying the findings in their manuscript fully available?

The** [**PLOS Data policy**](http://www.plosone.org/static/policies.action#sharing) **requires authors to make all data underlying the findings described in their manuscript fully available without restriction, with rare exception (please refer to the Data Availability Statement in the manuscript PDF file). The data should be provided as part of the manuscript or its supporting information, or deposited to a public repository. For example, in addition to summary statistics, the data points behind means, medians and variance measures should be available. If there are restrictions on publicly sharing data—e.g. participant privacy or use of data from a third party—those must be specified.

Reviewer #1: Yes

Reviewer #2: Yes**

**4. Is the manuscript presented in an intelligible fashion and written in standard English?

PLOS ONE does not copyedit accepted manuscripts, so the language in submitted articles must be clear, correct, and unambiguous. Any typographical or grammatical errors should be corrected at revision, so please note any specific errors here.

Reviewer #1: Yes

Reviewer #2: No**

5. Review Comments to the Author

**Please use the space provided to explain your answers to the questions above. You may also include additional comments for the author, including concerns about dual publication, research ethics, or publication ethics. (Please upload your review as an attachment if it exceeds 20,000 characters)

Reviewer #1: Overall, this is a useful descriptive study of reproductive health needs.**

**Abstract
States higher risk of STI. And pregnancy?**

We did ask questions on pregnancy but this is a topic for another paper. We wanted this paper to mainly focus on sexual health.
 **Intro
Can authors site what is known about repro risk in JJ youth?**

In Australia, a survey was conducted among juvenile justice youth detention in New South Wales. Of 19 young girls surveyed, 31.6% had ever been pregnant, first pregnancy was at 14.2 years. No questions were asked on abortions, miscarriages, stillbirths or drugs/smoking/alcohol while pregnant (Source: 2015. Young People in Custody Health Survey: Full Report. Justice Health & Forensic Mental Health Network and Juvenile Justice NSW. NSW Government: Malabar, Sydney.)

**Also, any hypothesized differences between kids with low levels of involvement vs longer-term incarceration**

Young people with lower levels of criminal justice involvement vs those in detention were associated with lower rates of ever having had sex (Chi square p<0.05). There were no differences between young people with medium levels of criminal justice involvement vs those in detention in terms of ever having had sex (Chi square p>0.05).

**Methods
Do not understand why approach necessary. Can authors clarify? Why were the selection criteria chosen?**

We selected young people aged 14-17 years old as there were very few sexual health and behavior studies conducted on young people below 16 years of age in Australia. Sexual health studies become more difficult to progress if the respondent is below 14 years of age due to issues with obtaining parental/guardian consent and also having to ensure that the respondent truly understands what they are volunteering for as a mature minor.

**Whose missing from the sample since its not representative?**

We can only compare age, sex and Indigenous status with other data from official government juvenile justice sources in Australia.

We did not compare the gender of this study population with other juvenile justice sources since we oversampled girls in our study for advanced statistical analysis (Note: 80% are boys under juvenile justice supervision). We compared this study population and age matched them to groups among juvenile defendants and youth justice populations in Queensland and Western Australia. We found that the proportion of young people, aged 14-15 years and 16-17 years in the MeH-JOSH study population, may differ by 2%-6% compared to their community based peers under official government juvenile justice supervision (Figure 1).

**Figure 1. Young people by age group under supervision on an average day by state and compared with MeH-JOSH study sample**

*Source: AIHW Youth Justice Qld S132a-S132c and WA S134a-S134c. Available at:* [*https://www.aihw.gov.au/getmedia/38637613-61ed-4709-a42a-1c1b77d8138d/aihw-juv-116-state-and-territory-tables-s128-to-s143-2016-17-data-table.xlsx.aspx*](https://www.aihw.gov.au/getmedia/38637613-61ed-4709-a42a-1c1b77d8138d/aihw-juv-116-state-and-territory-tables-s128-to-s143-2016-17-data-table.xlsx.aspx)

*Children’s Court of Qld Annual Report 2016-2017*

In general, the study had an over representation of Aboriginal (Indigenous) young people when we compared it to the general Aboriginal populations in Australia and reflects the greater involvement of this population in the justice system (Figure 2).

**Figure 2. Aboriginal populations in Australia and MeH-JOSH**

Aboriginal Populations in Australia

|  | Indigenous Population |
| --- | --- |
| QLD (10-17 years)* | 7.47277% |
| WA (10-17 years)* | 6.56638% |
| Australia (10-17 years)* | 5.41519% |
| Australia (All Ages)# | 3.30027% |
| 2018 SSASH (14-17 years)^ | 4.26019% |
| MeH-JOSH (14-17 years) | 43.6285% |

*Source: *AIHW Youth Justice. Table S31: Australian population aged 10–17 by Indigenous status, states and territories, December 2013 to December 2017. Available at:* [*https://www.aihw.gov.au/getmedia/993d8e77-b18e-4482-b9b9-12fe838db158/aihw-juv-128-youth-detention-population-in-Australia-2018-data-tables.xlsx.aspx*](https://www.aihw.gov.au/getmedia/993d8e77-b18e-4482-b9b9-12fe838db158/aihw-juv-128-youth-detention-population-in-Australia-2018-data-tables.xlsx.aspx)

*# Australian Bureau of Statistics (2016). 3238.0.55.001 – Estimates of Aboriginal and Torres Strait Islander Australians, June 2016. Available at: http://www.abs.gov.au/ausstats/abs@.nsf/Lookup/3238.0.55.001main+features1June%202016*

*^2018 Secondary School Student Sexual Health Survey, personal communication, C Fisher.*

**Exactly 42 mintues? seems awkward. suggest saying 40 min.**

42 minutes is more accurate

**consent process is stated for only 1 of the recruitment methods**

We have added to the manuscript:

“Due to the nature of this population and from our experiences of conducting surveys among young offenders in Australia[3], we expected that a high proportion of young people would not have a good relationship with their parent(s) or with the adults responsible for them. Thus human research ethics approval was sought and given for young people recruited in the survey to be treated as mature minors.[14] Nevertheless, all young people approached were asked by recruiters if we could contact their parents or guardians for permission to allow them to participate in the survey. Almost all respondents refused us contact or to give any contact details of their parents or guardians preferring to give consent themselves. Recruiters were required to administer a Gillick Competency checklist to ensure that all respondents met the criteria of a mature minor.”

**Discussion
First sentence very long**

Thank you for pointing this out. We have edited the sentence to make our points clearer.

“We determined that young people involved in the justice system are at greater risk for STIs for the following reasons: (i) they have a higher engagement in sex compared with their peers in school surveys (1.3 to 3.2 times higher); (ii) are often starting to have sex at a younger age (median age 14 years compared to 17 years in the general Australian population and 15 years in the general Indigenous population)^19^ ^20^; (iii) are significantly more likely to report large numbers of sexual partners (6+) at a young age; and (iv) around half were not using a condom during their last sexual encounter.”

**Do authors think youth recalling HPV vaccination correctly? Is never been vaccinated variable category based on records or youth recall?**

Memory recall is not altogether an accurate indicator but other studies in Australia also reveal similar results for similar questions on HPV (e.g. SSASH 2018), most do not recall if they had been vaccinated for HPV which is an interesting finding in itself. Perhaps in future studies we can ask respondents for their consent to match them up with their vaccination records.

**I'm not Austrialian. Familiar with Aboriginal but what's the significant of Torres Strait Islander? Perhaps explain briefly in Intro of Methods for international audience. I'm guessing these are also "indigenous" youth, but not sure.**

Torres Strait Islanders are also Indigenous in Australia. We have added the following:

“Young people who identified as Aboriginal and/or Torres Strait Islander (Indigenous) descent were overrepresented in the survey sample compared with the general population (44% versus 5%), reflecting the greater involvement of this population in the justice system.”

**Regarding disproportionate confinement, what is the prevalence of aboriginal and islanders on the island?**

According to the Australian Bureau of Statistics, there are 590,062 Aboriginal People and 32,344 Torres Strait Islanders in Australia. In addition, there are 26,767 who are both Aboriginal and Torres Strait Islanders.

<https://quickstats.censusdata.abs.gov.au/census_services/getproduct/census/2016/quickstat/IREG307?opendocument>

**Juvenile justice data

Limitations
Authors' frustration with approval process and resultant sampling framework comes through. perhaps revise**

We would like to retain this paragraph as it provides a rationale for the sampling framework.

“A purposive sampling design based on strict selection criteria was used to recruit young people in the community; participants must be aged between 14 and 17 years and had to have had contact with the criminal justice system in the past or present. This approach was necessary as we had been denied permission to conduct a cross-sectional representative survey of young people (both in detention and those serving community orders) from youth justice and corrective services departments in Queensland and Western Australia.”

**I would like to see the authors expand on differences between kids of varying levels of contact. Did they do sub-analyses? At the very least, Discussion should comment on this. Could be added as a limitation**

We did not conduct any subanalysis as we wanted this first paper to be descriptive.

**Table
Suggest title column with name that clearly indicates that MEH group is the JJ group**

Technically, some aren’t under juvenile justice supervision but under supervision of the police etc. We have added, “**MEH-JOSH (JUSTICE INVOLVED YOUNG PEOPLE)”**

**Formatting of tables could be improved. Suggest n and then % in parenthesis (unless authors are following PLOS table formatting guidelines that I am unaware of). It will make table more digestible.**

It becomes harder for us to edit n (%) as they are not in separate columns. We leave this to the editor to decide. This format has been accepted in our other publications.

**Footnotes should briefly explain what the different samples are.**

Added to the tables:

^1^ 2014 YMM – 2014 Young Minds Matter: 2nd Survey of the Mental Health of Australian Children Survey

^2^ 2018 SSASH – 6th National Survey of Secondary Students and Sexual Health

**What is the rationale for the bolding? It seems inconsistent.**

Fixed

**Reviewer #2: This study offered a descriptive comparison of sexual behaviors among justice-involved and community youth in Australia. The authors interviewed adolescents currently or previously involved with the criminal justice system, and compared their responses to non-delinquent peers using alternative data sources. Youth in the justice-involved sample were more likely to engage in sex and start having sex at a younger age, and were more likely to report having 6 or more partners.

Overall, I think the premise of this paper is a useful contribution. While researchers argue that justice-involved youth are at heightened risk of sexual risk-taking, there are few formal comparisons with nationally representative samples to empirically support this assertion. Although this paper provides evidence for these assumptions, there are a number of substantial issues that would need to be addressed prior to publication.**

**Major issues

1. The authors could improve the introduction by making a stronger argument as to why delinquent populations may be at risk for sexual risk-taking. Right now, it seems as though their argument rests on the idea that delinquent populations may not be reached by traditional intervention efforts, and ignores the theoretical and empirical work that suggests that the same factors that put youth at risk for delinquency may drive their tendency to engage in riskier sexual behaviors. I would recommend reviewing a number of articles:**

**a. Robbins, R. N., & Bryan, A. (2004). Relationships between future orientation, impulsive sensation seeking, and risk behavior among adjudicated adolescents. Journal of adolescent research, 19(4), 428-445.
b. Bryan, A. D., Schmiege, S. J., & Magnan, R. E. (2012). Marijuana use and risky sexual behavior among high-risk adolescents: trajectories, risk factors, and event-level relationships. Developmental psychology, 48(5), 1429.
c. In general, Angela Bryan has a number of relevant articles on this subject that are worth reading.**

Thank you. We have added the paragraph,

“Studies have suggested that drug addiction and mental health disorders that put youth at risk for offending may also drive their tendency to engage in riskier sexual behaviours.^4-7^ One study reported increased sexual risk behaviours among juvenile detainees compared to the general population.^8^ Formal comparisons of young offenders with community populations and their sexual health and behaviours are extremely limited.^9^”

**2. Similarly, although formal comparisons with community populations are limited, there are a few studies that address a similar subject matter that seem like they should be included:**

**a. Elkington, K. S., Teplin, L. A., Mericle, A. A., Welty, L. J., Romero, E. G., & Abram, K. M. (2008). HIV/sexually transmitted infection risk behaviors in delinquent youth with psychiatric disorders: A longitudinal study. Journal of the American Academy of Child & Adolescent Psychiatry, 47(8), 901-911.
b. Teplin, L. A., Mericle, A. A., McClelland, G. M., & Abram, K. M. (2003). HIV and AIDS risk behaviors in juvenile detainees: Implications for public health policy. American Journal of Public Health, 93(6), 906-912.
c. Teplin, L. A., Elkington, K. S., McClelland, G. M., Abram, K. M., Mericle, A. A., & Washburn, J. J. (2005). Major mental disorders, substance use disorders, comorbidity, and HIV-AIDS risk behaviors in juvenile detainees. Psychiatric Services, 56(7), 823-828.
d. DiClemente, R. J., Lanier, M. M., Horan, P. F., & Lodico, M. (1991). Comparison of AIDS knowledge, attitudes, and behaviors among incarcerated adolescents and a public school sample in San Francisco. American journalof public health,81(5), 628–630.**

Thank you. We have added the paragraph,

“Studies have suggested that drug addiction and mental health disorders that put youth at risk for offending may also drive their tendency to engage in riskier sexual behaviours.[4-7] One study reported increased sexual risk behaviours among juvenile detainees compared to the general population.[8] Formal comparisons of young offenders with community populations and their sexual health and behaviours are extremely limited.[9]”

**3. The authors should provide more information on what they mean by a “purposive sampling design.” The authors do not explain what the calculated sample size was, which is odd given the heading of the section.**

We have edited the paragraph to read more accurately,

“A purposive sampling design based on strict selection criteria was used to recruit young people in the community; participants must be aged between 14 and 17 years and had to have had contact with the criminal justice system in the past or present. This approach was necessary as approval was not granted to conduct a cross-sectional representative survey of young people (both in detention and those serving community orders) from youth justice and corrective services departments in Queensland and Western Australia.

Quota sample sizes were calculated based on known demographic characteristics of the Australian juvenile offender population (age and gender).[10] Aboriginal young people in this study were over represented (44%) compared to 5% in the general Australian population[13] and reflects the greater involvement of this population in the justice system. In Australia, the majority of young people under supervision on an average day in 2017–18 were male (81%).[10] Females were deliberately oversampled in this study to enable more advanced statistical analysis”

**4. The authors should offer some information as to if the sample differed across the four different recruitment methods, given that they are ultimately combined.**

We added the following sentence,

Site of recruitment was not a confounding factor for age and gender although it was significant for Aboriginality (p<0.05).

**5. The brevity of the methods makes it difficult to discern how the authors measured sexual behavior. Many of the terms used are quite broad (justice system involvement, sexual history, sexual knowledge) and could encompass a wide variety of topics. The authors should expand on this section and provide more details as to what their measures asked, providing specific examples of the questions.**

We did not list the survey questions as there is a word limit for original papers. Nevertheless, the survey questions and measures are listed in the attached tables.

**6. The authors describe the comparison sample under “Data Analysis” but it seems like this information should be included in the methods section, in the same section where the justice-system sample is described. We are also given very few details about these comparison samples and some additional information on these separate studies would be helpful, as well as an explanation of why two different studies were employed. If the two studies included different variables, then the authors should describe the different measures covered in each study.**

We have added the following sentence to make it clearer on how the variables were selected.

“Descriptive statistics were generated using Statistics SPSS 25 from original datasets of the MeH-JOSH survey and age matched with the Young Minds Matter: 2^nd^ Survey of the Mental Health of Australian Children Survey (YMM), a probability sample of young people aged between 4 and 17 years old from 5,500 randomly sampled families in Australia.[15] Separate age matched data analysis was provided by C. Fisher for the 6^th^ National Survey of Secondary Students and Sexual Health (SSASH), a convenience sample of adolescents in the community that had agreed to take part in an online survey in Australia. [16].

The self-reported sexual health and behaviours of participants in our survey were compared with their age-matched peers in the YMM[15] and SSASH[16] surveys. Sexual identity proportions were compared using the Chi-squared test. MeH-JOSH sexual health and behavioural questions for this paper were selected based on the equivalence or sameness of questions from the YMM and SSASH survey (for example, MEH-JOSH – “Can you tell me if you have ever had sex?” YMM and SSASH – “Have you ever had sexual intercourse?”).”

**Similarly, there is very little information provided on what method of matching was used. It also seems as though the authors only matched on age, and it is unclear if other characteristics (gender, SES, race/ethnicity) were considered in the matching process. Given the possible differences in the two populations (justice involved vs. nationally representative), it is possible that these differences could explain different rates of sexual risk-taking, beyond justice-system involvement.**

We did not match for gender as we know from current youth justice statistical data that juvenile offender populations were mostly male (80%) and >50-80% Indigenous. We have added an extra row in Table 1 to include gender proportions in the different studies. Chi2 tests showed that gender and ever having had sex was not significant (p>0.05) in the MeH-JOSH, YMM and SSASH surveys. Drug use, alcohol abuse and mental health disorders, however, were associated with ever having sex in the MeH-JOSH study but this is for another paper.

**7. The results appeared a bit disorganized, and as a result were difficult to follow. First, I think it would be helpful for the authors to build on their analytic plan in the Data Analysis section. Although they note the use of Chi Square tests, it would be helpful to explain which variables they are comparing (and in which samples) before jumping into the results.**

We edited the sentence to be clearer,

“Sexual identity proportions were compared using the Chi-squared test.”

**The first paragraph under “Sexual health and behaviours” seems like it should be earlier in the methods, not in the results. A similar set of information on the two nationally representative samples would also be helpful.**

Descriptive epidemiological papers usually include demographics of the survey population in the results section as a standard.

**a. The authors report differences in sexual orientation by gender, but it is not clear 1) what sample this is referring to (justice involved, nationally representative, both combined) and 2) why a gender comparison was made. It does not seem like this was part of the goal of the paper, and the authors did not make any comparisons between the different samples. It is evident from Table 2 that this information is just referring to the justice-involved sample, but it is not clear why no formal comparisons were made with the SSASH sample.**

We have added clarification on which study it was from in the sentence.

“Of the participants, 91% identified as heterosexual, 6% bisexual, and 1% gay or homosexual/lesbian. Young women in the MeH-JOSH study were more likely to identify as bisexual/homosexual/queer than young men, (17% versus 2%, χ²(1)>=28.75, *p<.001*). Similarly, young women in the SSASH study were also more likely to identify as non-heterosexual (27.6% vs 21.2%, *X*^2^(1)=36.35, *p<0.001*) (cf. C Fisher). Thirty-one per cent of respondents reported they were in a current relationship, either dating or living with their partner. Among 14-15 year old and 16-17 year old age groups, 27% and 34% respectively, were in a relationship at the time of the interview. (Table 2)”

**b. The next paragraph, “Of the total sample, 76%...” also needs to be clarified. It would be easier to interpret if the authors provided the percentages for both groups, rather than providing a percentage and then stating it was 1.3-3.2x higher than the other groups. I’m also not sure what the next sentence (“However, this was lower than the 92%...) refers to. Based on Figure 1 it looks like these analyses were split by age, but this is not clear from the methods or results. In addition, it is not clear if the original item combined oral, vaginal and/or anal sex, or if this was combined by the authors.**

We have edited the paragraph to read more clearly,

“Of the total MeH-JOSH sample, 76% (n=348) reported having ever had oral, vaginal and/or anal sex, which was higher than among school aged children in the SSASH (57%) and YMM (24%) surveys (Fig 1 and Table 2). In the MeH-JOSH survey, 74% (n=338) had had vaginal and/or anal sex compared to 45% in the SSASH survey (Figure 1 and Table 2). We noted that these figures were lower than the 92% sexual experience reported elsewhere among young people under the age of 16 years in New South Wales juvenile detention centres (Fig 1).[2] Condom use at the last sexual encounter was 55% for both MeH-JOSH and SSASH respondents compared to 64% among YMM respondents (Table 3)”

**It would be preferable to look at these types of sexual behaviors separately, if possible.**

In Table 3, we looked at these sexual behaviours (oral, vaginal and anal sex) separately.

**c. The next section (“Of the 348 who reported…) also lacks organization. It would be helpful to provide comparable information across samples, and then provide a formal comparison using a statistical test. For example, 76% of youth in the justice-sample reported having sex for the first time at age 14 or younger. What is the comparable statistic for the other sample?**

The YMM survey did not ask about age of sexual initiation and the SSASH survey collected age of sexual initiation as a categorical rather than a continuous variable and thus we cannot compare the data.

**The authors instead provide some descriptive information about the justice-sample, but then offer only a select number of comparisons (e.g., how oral sex was less common and vaginal sex was more common among justice-system participants). There is also no formal statistic provided to show whether these differences are statistically significant.**

**d. The authors indicate that justice-system youth initiated sexual activity at an earlier age, but it is not clear if this was formally tested. What statistical test did they use?**

The YMM survey did not ask age of sexual initiation and the SSASH survey collected age of sexual initiation as a categorical rather than a continuous variable and thus we cannot compare or test the data.

We can only discuss our data with a longitudinal study of Australian adults who recalled their first sexual initiation at 17 years of age.

17.	Rissel C, Heywood W, de Visser RO, Simpson JM, Grulich AE, Badcock PB, et al. First vaginal intercourse and oral sex among a representative sample of Australian adults: the Second Australian Study of Health and Relationships. Sex Health. 2014; 11:406-15.

**e. For sexual initiation, it is confusing to say 41% of justice-involved participants reported 6+ partners, which was 2.6x higher than the comparison sample. It would be helpful just to provide the percentage for the other samples as well.**

We have edited the sentence to read,

“Of those who have had sex, 41% of justice-involved MeH-JOSH participants reported having six or more sexual partners in their lifetime compared to 16% among school aged children in the YMM survey, and higher at all ages (Figure 3).”

**f. The last section (“Of the 348 sexually experienced participants, 37%...”), it appears the authors only provide descriptive information for the justice involved sample. Without a formal comparison it is difficult to discern if these percentages are low, high or average.**

We have no formal comparisons but we reported it for any future studies to compare STI rates in similar populations.

**g. Overall, the results would need to be entirely restructured to offer descriptive statistics for all samples, and then provide formal comparisons using the appropriate statistical tests.**

Around 76% of young respondents in the MeH-JOSH study are already having sex so to compare them ever having sex with other variables (gender and age) may be self-fulfilling.

**8. The discussion does not entirely map onto the results from the paper. For example, the authors argue justice system youth are at greater risk for STIs, but this ignores the finding that their condom use is comparable to the community samples.**

Condom use was for their last sexual encounter rather than lifetime sexual encounter. We cannot discount other high risk sexual practices.

**The authors also argue that most had not been tested for HPV, but we have no comparison to the community sample to know if this is something that is particular to justice system populations. It seems like the primary finding is that justice system youth are more likely to have had sex, but given that all sex is not inherently risky, it seems like some of these other statistics are worth discussing (condom use, HPV testing, HIV testing).**

We have no formal comparisons but we reported it for any future studies to compare reported HPV vaccination rates in similar populations.

**Minor issues
1. On page 4, it should read, “The Mental Health, Sexual Health and Reproductive…study aimed to describe” (rather than describes).**

Fixed

**2. On the top of page 5, “The main aim of the survey was…” (not were).**

Fixed

**3. The authors switch back and forth between saying justice involved vs. using the MeH-JOSH acronym. It would be helpful if this was consistent.**

Fixed

**4. Table 2 is overwhelming to look at, and could be broken down into smaller pieces for easier interpretation.**

 We have now separated this into two tables: Table 3 and Table 4.

6. PLOS authors have the option to publish the peer review history of their article ([what does this mean?](https://journals.plos.org/plosone/s/editorial-and-peer-review-process#loc-peer-review-history)). If published, this will include your full peer review and any attached files.


If you choose “no”, your identity will remain anonymous but your review may still be made public.


**Do you want your identity to be public for this peer review?** For information about this choice, including consent withdrawal, please see our [Privacy Policy](https://www.plos.org/privacy-policy).

Reviewer #1: No

Reviewer #2: No


[NOTE: If reviewer comments were submitted as an attachment file, they will be attached to this email and accessible via the submission site. Please log into your account, locate the manuscript record, and check for the action link "View Attachments". If this link does not appear, there are no attachment files to be viewed.]


While revising your submission, please upload your figure files to the Preflight Analysis and Conversion Engine (PACE) digital diagnostic tool, <https://pacev2.apexcovantage.com/>. PACE helps ensure that figures meet PLOS requirements. To use PACE, you must first register as a user. Registration is free. Then, login and navigate to the UPLOAD tab, where you will find detailed instructions on how to use the tool. If you encounter any issues or have any questions when using PACE, please email us at [figures@plos.org](mailto:figures@plos.org). Please note that Supporting Information files do not need this step.

*In compliance with data protection regulations, you may request that we remove your personal registration details at any time.* [*(Remove my information/details)*](https://www.editorialmanager.com/pone/login.asp?a=r)*. Please contact the pub*
