# Supplementary material for: The sexual behaviours of adolescents aged between 14 and 17 years involved with the juvenile justice system in Australia: A community-based survey
Source: PLoS One. 2020 Dec 28;15(12):e0243633. doi: 10.1371/journal.pone.0243633 (PMC7769256; doi:10.1371/journal.pone.0243633)
Supplement: Attachment — Submitted filename: PLoS One Response to reviewers final.docx [file pone.0243633.s003.docx]

Dear Managing Editor and Reviewers,

We thank you for your consideration of our manuscript and for the detailed feedback that has been provided. We have also addressed Reviewer #1’s comments from the first round again as requested.

Substantial changes have been made to the manuscript which we believe has greatly enhanced its rigour and suitability for publication. Please see our responses to specific comments below. We look forward to hearing your response.

Kind regards,

Tony Butler

**Editor Comments (if provided):**

Although this manuscript remains a timely and topical piece, many of the concerns expressed by the reviewers have not been addressed. Reviewer #2 has reviewed the revised manuscript, and outlined the persistent issues in the additional review below. Reviewer #1 was not available for repeat review, but on my reading of the responses I have identified several issues. Many of the critiques were addressed with comments in the response, but no changes were actually made in the manuscript. For example, the request to incorporate some discussion of what is known about about reproductive health risk in this population was clearly intended to motivate a change to the manuscript, not just a sentence about the available literature. The clarifications regarding the sampling frame were also intended to be reflected in the manuscript, and the question about participant recall of HPV vaccination addressed in the discussion or some other place. Please review again the critiques from Reviewer #1, and address each in the manuscript. These substantive changes are necessary to meet the publication criteria for PLOS ONE.

**Reviewer #1:** Overall, this is a useful descriptive study of reproductive health needs.

**Comment:** Abstract
States higher risk of STI. And pregnancy?

**Response:** We did ask questions on pregnancy and reproductive health but this will be covered in a separate paper. We wanted this paper to focus on the findings of sexual health and sexual behaviours.

**Comment:** Intro
Can authors site what is known about repro risk in JJ youth?

**Response:** We have added to the manuscript:

“In Australia, a survey was conducted among juvenile justice youth detention in New South Wales. Of 19 young girls surveyed, 31.6% had ever been pregnant, first pregnancy was at 14.2 years. No questions were asked on abortions, miscarriages, stillbirths or drugs/smoking/alcohol while pregnant.[3]”

**Comment:** Also, any hypothesized differences between kids with low levels of involvement vs longer-term incarceration

**Response:** We have added to the manuscript in the Discussion:

“Since this was an exploratory study, we did not hypothesise about differences between the incarcerated youth justice population and those with less involvement. Differences stratified by involvement with the justice system can be explored in future.”

**Comment:** Methods

Do not understand why approach necessary. Can authors clarify? Why were the selection criteria chosen?

**Response:** We have added to the manuscript to Sampling and sample size:

“A sample of young people aged 14-17 years old was selected to reflect the age of the youth justice population in Australia. Those over 17 (that is, 18 or older) are considered adults. Ethical issues prevented interviewing those below 14 years, such as obtaining parental/guardian consent, and also having to ensure that very young respondents could truly understand what they would be consenting to.“

**Comment:** Whose missing from the sample since its not representative?

**Response:** The aim of the study was not to generate a statistically representative sample of all justice-involved young people in QLD and WA (a group for which there is not an obvious sampling frame from which a random sample could be drawn) but to explore the sexual health and behaviours in a diverse range of young people across the spectrum of justice involvement.

We note that this is one of few studies, and the only recent study, to examine the sexual behaviours amongst this population, which is due to the practical and methodological difficulties of robustly surveying this group of young people.

Regarding the generalisability of the findings, we have added to the manuscript in the Discussion:

“Limitations of this study include the use of a non-random sampling strategy (necessary to overcome practical difficulties of reaching such individuals), resulting in a sample that may not be statistically representative of the target population (i.e. the full range of justice-involved young people).”

**Comment:** Exactly 42 mintues? seems awkward. suggest saying 40 min.

**Response:** Agree, this has been changed to approximately 40 minutes.

**Comment:** consent process is stated for only 1 of the recruitment methods

**Response:** We have added the following text to explain the consent process as part of a separate section Consent:

“Due to the nature of this population and from our experiences of conducting surveys among young offenders in Australia[3], the study expected that a high proportion of young people would not have a good relationship with their parent(s) or with the adults responsible for them. Thus human research ethics approval was sought and given for young people recruited in the survey to be treated as mature minors.[14] Nevertheless, all young people approached outside the courts were still asked by recruiters if we could contact their parents or guardians for permission to allow them to participate in the survey. Almost all respondents refused us contact or to give any contact details of their parents or guardians preferring to give consent themselves. Recruiters were required to administer a Gillick Competency checklist to ensure that all respondents met the criteria of a mature minor.”

**Comment:** Discussion
First sentence very long

**Response:** Thank you for pointing this out. We have edited the sentence to make our points clearer.

“Young people involved in the justice system have a higher engagement in sex compared with their peers in school surveys (1.3 to 3.2 times higher), are often starting to have sex at a younger age (median age 14 years compared to 17 years in the general Australian population and 15 years in the general Indigenous population)[17] [18], and are significantly more likely to report large numbers of sexual partners (6+) at a young age. While similar rates of young people used a condom during their last sexual encounter across MeH-JOSH, SSASH and YMM, engagement in more frequent and riskier sexual behaviours suggests that this may pose more of a STI risk to justice-involved young people.

**Comment:** Do authors think youth recalling HPV vaccination correctly? Is never been vaccinated variable category based on records or youth recall?

**Response:** We have added to the manuscript in the Discussion:

“Findings are based on self-report and may be impacted by recall or social desirability bias. While memory recall is not altogether an accurate indicator other studies in Australia also reveal similar results for similar questions on HPV (e.g. SSASH 2018), most do not recall if they had been vaccinated for HPV which is an interesting finding in itself. Perhaps in future studies we can ask respondents for their consent to match them up with their vaccination records.”

**Comment:** I'm not Austrialian. Familiar with Aboriginal but what's the significant of Torres Strait Islander? Perhaps explain briefly in Intro of Methods for international audience. I'm guessing these are also "indigenous" youth, but not sure.

**Response:** Torres Strait Islanders are also Indigenous in Australia. We have added “(Indigenous)” to clarify in the manuscript:

“Young people who identified as Aboriginal and/or Torres Strait Islander (Indigenous) descent were overrepresented in the survey sample compared with the general population (44% versus 5%), reflecting the greater involvement of this population in the justice system.”

**Comment:** Regarding disproportionate confinement, what is the prevalence of aboriginal and islanders on the island?

**Response:** We have clarified in our manuscript:

“Aboriginal and/or Torres Strait Islander (Indigenous) young people in this study sample were over represented (44%) compared to 5% in the general Australian population and this reflects the greater involvement of this population in the justice system.[13]”

**Comment:** Juvenile justice data

Limitations
Authors' frustration with approval process and resultant sampling framework comes through. perhaps revise

**Response:** We have revised this in the manuscript:

“A purposive sampling design based on strict selection criteria was used to recruit young people in the community; participants must be aged between 14 and 17 years and had to have had contact with the criminal justice system in the past or present. This approach was necessary as permission was not granted to conduct the survey with young people in detention and those serving community orders.”

**Comment:** I would like to see the authors expand on differences between kids of varying levels of contact. Did they do sub-analyses? At the very least, Discussion should comment on this. Could be added as a limitation

**Response:** We have added to the manuscript in the Discussion:

“Since this was an exploratory study, we did not hypothesise about differences between the incarcerated youth justice population and those with less involvement. Differences stratified by involvement with the justice system can be explored in future.”

**Comment:** Table
Suggest title column with name that clearly indicates that MEH group is the JJ group

**Response:** To clarify, we have edited the table titles as follows:

**Comment:** Formatting of tables could be improved. Suggest n and then % in parenthesis (unless authors are following PLOS table formatting guidelines that I am unaware of). It will make table more digestible.

**Response:** Fixed.

**Comment:** Footnotes should briefly explain what the different samples are.

**Response:** Added to the tables:

^1^ 2014 YMM – 2014 Young Minds Matter: 2nd Survey of the Mental Health of Australian Children Survey

^2^ 2018 SSASH – 6th National Survey of Secondary Students and Sexual Health

**Comment:** What is the rationale for the bolding? It seems inconsistent.

**Response:** Fixed.

*[Note: HTML markup is below. Please do not edit.]*

*Reviewers' comments:

Reviewer's Responses to Questions*

*Comments to the Author

1. If the authors have adequately addressed your comments raised in a previous round of review and you feel that this manuscript is now acceptable for publication, you may indicate that here to bypass the “Comments to the Author” section, enter your conflict of interest statement in the “Confidential to Editor” section, and submit your "Accept" recommendation.*

*Reviewer #2: (No Response)*

*2. Is the manuscript technically sound, and do the data support the conclusions?

The manuscript must describe a technically sound piece of scientific research with data that supports the conclusions. Experiments must have been conducted rigorously, with appropriate controls, replication, and sample sizes. The conclusions must be drawn appropriately based on the data presented.*

*Reviewer #2: (No Response)*

*3. Has the statistical analysis been performed appropriately and rigorously?*

*Reviewer #2: (No Response)*

*4. Have the authors made all data underlying the findings in their manuscript fully available?

The* [*PLOS Data policy*](http://www.plosone.org/static/policies.action#sharing) *requires authors to make all data underlying the findings described in their manuscript fully available without restriction, with rare exception (please refer to the Data Availability Statement in the manuscript PDF file). The data should be provided as part of the manuscript or its supporting information, or deposited to a public repository. For example, in addition to summary statistics, the data points behind means, medians and variance measures should be available. If there are restrictions on publicly sharing data—e.g. participant privacy or use of data from a third party—those must be specified.*

*Reviewer #2: (No Response)*

*5. Is the manuscript presented in an intelligible fashion and written in standard English?

PLOS ONE does not copyedit accepted manuscripts, so the language in submitted articles must be clear, correct, and unambiguous. Any typographical or grammatical errors should be corrected at revision, so please note any specific errors here.*

*Reviewer #2: (No Response)*

*6. Review Comments to the Author

Please use the space provided to explain your answers to the questions above. You may also include additional comments for the author, including concerns about dual publication, research ethics, or publication ethics. (Please upload your review as an attachment if it exceeds 20,000 characters)*

**Reviewer #2:** The author's response is helpful and the changes largely address my original comments. A few things to consider: **Comment:** I think including the Chi Square comparisons sets the reader up to think other formal comparisons will be made. I understand this is a descriptive paper, but given no other gender comparisons were made, it seems a bit arbitrary. It would be helpful to understand why these specific comparisons were included, whereas other gender comparisons were not.

**Response:** Agreed. Gender comparisons were analysed for all variables and all tables have included these *p*-values. We have added to the manuscript:

“The self-reported sexual health and behaviours of participants in the MEH-JOSH survey (including sexual identity and attraction, relationship status, ever having sex (any, vaginal, anal, oral), sexual initiation, lifetime sexual encounters, last sexual encounter, STI testing and diagnosis, HPV vaccination, and willingness to have HPV vaccine) were compared with their age-matched peers in the YMM[15] and SSASH[16] surveys where applicable. Chi-squared tests were used to analyse gender differences in these variables in the MEH-JOSH survey. A test of two proportions was used to analyse gender differences in types of offences committed.”

The following gender comparison findings were added to the Participants section (please see Participants section in the manuscript to see full integration of these findings):

- There was a higher proportion of males than females in the 16-17 age group (56% versus 44%, χ²(1)=5.683, *p*=.017).
- A higher proportion of males were on a current sentence or order compared to females (40% versus 30%, χ²(1)=4.567, *p*=.033).

The following gender comparison findings were added to the Results section (please see Results section in the manuscript to see full integration of these findings):

- Young females in the MeH-JOSH study were more likely to identify as bisexual than young males, (15% versus 1%, χ²(3)=34.295, *p<*.001), and this is also reflected in their sexual attraction (14% versus 3%, χ²(3)=20.031, *p*<.001). This is similar to the SSASH study whereby young females in the general population were also more likely to identify as bisexual (C Fisher, personal communication, September 2020).
- Justice-involved young females were more likely to have had sex with a person 18 years or older for their first sexual partner (13% versus 3%, χ²(1)=12.056, *p*=.001), and their last sexual partner (32% versus 12%, χ²(1)=19.812, *p<*.001), compared to males.
- Justice-involved young males were more likely to report numbers of 6 or more sexual partners than females (45% versus 34%, χ²(1)=4.091, *p*=.043).
- For their last sexual encounter, justice-involved young females were more likely to have sex with their current girlfriend/boyfriend (54% versus 40%), while males were more likely to have sex with a stranger (16% versus 4%, χ²(3)=13.629, *p*=.003). Young males were more likely to use a condom during their last sexual encounter than females (59% versus 48%, χ²(1)=4.094, *p*=.043).
- Of the 348 sexually experienced participants, 37% (n=129) reported prior testing for sexually transmissible infections (STIs) with young females more likely to have been tested recently in the last year compared to males (44% versus 24%, χ²(2)=9.392, p=.009). Young females were more likely to be tested by a general practitioner (57% versus 40%), while males were more likely to be tested in a juvenile detention centre or prison (20% versus 3%, χ²(5)=19.377, p=.002).
- Young females were more likely to have been HPV vaccinated than males (38% versus 26%, χ²(2)=7.361, *p*=.025)*.*
- A higher proportion of young females than males indicated a willingness to be HPV vaccinated (68% versus 53%, χ²(2)=6.988, *p*=.030).

**Comment:** On page 8 the authors also distinguish between the 14-15 YO and 16-17 YOs in regard to relationship status, but I don't see this separated in Table 2 anywhere.

**Response:** Well-spotted. We have modified the sentence to remove the reference to Table 2 here in our manuscript as follows:

**“**Among 14-15 year old and 16-17 year old age groups, 27% and 34% respectively, were in a relationship at the time of the interview.”

**Comment:** I still have some concerns that the authors may be overstating findings since formal comparisons were not possible. For example, on page 10 the authors state that "We determined that young people involved in the justice system are at great risk for STIS for the following reasons...around half were not using a condom during their last sexual encounter." As I brought up previously, the rates for the other samples were also not that high, and this phrasing makes it seem as though the justice sample is at a statistically higher risk for STI partly due to condom use (compared to the other samples). I think it would reflect the findings more accurately to explain the findings more descriptively, rather than using language that makes it seem formal comparisons were made.

**Response:** We agree with this concern of overstating findings, and have modified the language in our manuscript as follows:

“We have observed that young people involved in the justice system have a higher engagement in sex compared with their peers in school surveys (1.3 to 3.2 times higher), are often starting to have sex at a younger age (median age 14 years compared to 17 years in the general Australian population and 15 years in the general Indigenous population)[17] [18], and are significantly more likely to report large numbers of sexual partners (6+) at a young age. While similar rates of young people used a condom during their last sexual encounter across MeH-JOSH, SSASH and YMM, engagement in more frequent and riskier sexual behaviours suggests that this may pose more of a STI risk to justice-involved young people.”

“The study demonstrates how young people involved in the justice system may be at high risk of STIs and that it is possible to target this usually hard to access population through youth oriented community spaces without juvenile justice or corrective services involvement.”

Also, we have modified the language in our abstract:

“The sexual behaviours of young people involved in the justice system in this study suggest they may be at a greater risk for sexually transmissible infections than their age-matched peers in the general population.”

**Comment:** It seems reasonable to at least include a limitation in the discussion, explicitly stating that formal statistical comparisons could not be made across samples.

**Response:** We agree. We have added to the manuscript in the Discussion:

“Another limitation of this study is that it was not possible to conduct formal comparisons across these different survey samples.”

*7. PLOS authors have the option to publish the peer review history of their article (*[*what does this mean?*](https://journals.plos.org/plosone/s/editorial-and-peer-review-process#loc-peer-review-history)*). If published, this will include your full peer review and any attached files.


If you choose “no”, your identity will remain anonymous but your review may still be made public.


Do you want your identity to be public for this peer review? For information about this choice, including consent withdrawal, please see our* [*Privacy Policy*](https://www.plos.org/privacy-policy)*.*

*Reviewer #2: No*

*[NOTE: If reviewer comments were submitted as an attachment file, they will be attached to this email and accessible via the submission site. Please log into your account, locate the manuscript record, and check for the action link "View Attachments". If this link does not appear, there are no attachment files.]

While revising your submission, please upload your figure files to the Preflight Analysis and Conversion Engine (PACE) digital diagnostic tool,* [*https://pacev2.apexcovantage.com/*](https://pacev2.apexcovantage.com/)*. PACE helps ensure that figures meet PLOS requirements. To use PACE, you must first register as a user. Registration is free. Then, login and navigate to the UPLOAD tab, where you will find detailed instructions on how to use the tool. If you encounter any issues or have any questions when using PACE, please email PLOS at* [*figures@plos.org*](mailto:figures@plos.org)*. Please note that Supporting Information files do not need this step.*
